# Public health management of group A streptococcal infection in mother-baby pairs in England; a case series review

**DOI:** 10.1186/2047-2994-4-S1-P107

**Published:** 2015-06-16

**Authors:** SJ Howard, K Stoker, K Foster

**Affiliations:** 1 North East Health Protection Team, Public Health England, Newcastle upon Tyne, UK

## Introduction

Group A streptococci (GAS) are causative organisms in a large and increasing proportion of UK puerperal sepsis deaths, and remain an important consideration in neonatal sepsis in the UK and around the world. UK guidelines advise that both mother and baby should be treated with antibiotics if either develops an invasive GAS (iGAS) infection in the 28 days following birth to reduce the risk of iGAS developing in the other party.

## Objectives

To assess compliance with the UK guidelines in mothers who develop GAS infection in the puerperium over a 2-year period across the North East of England.

## Methods

We reviewed records of all cases of GAS infection notified to the North East Health Protection Team (part of Public Health England) between 1 Sep 2012 and 31 Aug 2014 in women who had given birth in the preceding 28 days. We assessed whether antibiotics had been prescribed in each case, and the process through which antibiotic prescription had been arranged.

## Results

In 19 of 24 pairs, both mother and baby received antibiotics, though 2 babies received courses which deviated from the guidance. The hospital treating the mother prescribed antibiotics for the baby in 14 pairs, though agreed to do so only after consultant intervention in 3 of these pairs. GPs prescribed antibiotics for 5 remaining treated babies.

In 2 pairs, only the mother received antibiotics.

In 3 pairs, neither mother nor baby received antibiotics.

## Conclusion

Some variation is explained by the clinical picture: there is little rationale to treat mothers (or babies of mothers) who have GAS isolated on microbiological samples but whose clinical picture does not suggest iGAS infection.

Some variation resulted from poor understanding: 2 babies were not prescribed antibiotics as they were assessed as "clinically well", missing the point of antibiotic prophylaxis. Some junior staff refused to prescribe antibiotics until instructed to do so by a consultant.

To improve consistency of practice, the Health Proteciton Team is developing a regional protocol for treatment of puerperal GAS cases in conjunction with clinical colleagues.

## Disclosure of interest

None declared.
